# Spectroscopic Studies of Baltic Amber—Critical Analysis

**DOI:** 10.3390/molecules30122617

**Published:** 2025-06-17

**Authors:** Mirosław Kwaśny, Aneta Bombalska

**Affiliations:** Institute of Optoelectronics, Military University of Technology, gen. S. Kaliskiego 2, 00-908 Warsaw, Poland; miroslaw.kwasny@wat.edu.pl

**Keywords:** Baltic amber, amber aging, fluorescence, FTIR

## Abstract

Using optical spectroscopy methods including absorption in the UV-VIS, FTIR, Raman, and fluorescence, the spectra of 25 different Baltic amber samples were measured, and the ability of each method to distinguish between thermally modified and naturally aged material was analyzed. The natural ambers studied are characterized by a wide range of spectral properties: the position of the transmission edge in the UV-VIS spectra, the absorbance ratios of the C-H and C=O groups in the IR spectra, a difference of approximately 20% in the fluorescence intensity level, and differences in the band ratios in the C=C and C-H bonds in the Raman spectrum. Spectral studies were carried out on samples of natural and thermally modified amber at temperatures of 100, 150, and 200 °C for 2–8 h. Drastic changes occur at temperatures above 150 °C: the color changes to dark brown, the UV-VIS transmission edge shifts, the absorbance of the C=O group increases, the absorbance intensity of the C=C bond decreases, and fluorescence disappears. In some special cases, fluorescence methods allow for the unambiguous distinction of amber from different geographical regions (e.g., Baltic and Dominican). Spectroscopic methods can distinguish natural amber from thermally modified amber only for large changes in the spectrum at temperatures of 150–200; for smaller changes, the differences between individual samples of natural amber may be greater than in the case of thermal modification.

## 1. Introduction

Natural Baltic amber (succinite) is the most common fossil resin on earth, constituting as much as 90% of all fossils, and is one of the most common decorative stones. Its unambiguous identification is therefore very important in archeology, chemistry, paleontology, and mineralogical and gemological sciences [1]. It was formed more than 40 million years ago from amber-bearing trees (*Pseudolarix wehri*) found in the Fennoscandia Forest (now Scandinavia) [2].

Succinite occurs in the Gdańsk Delta, where the world’s largest amber mine (Primorskoje) is located (Kaliningrad Oblast, Russia Federation). Succinite deposits also occur in northern and southeastern Poland (Parczew Delta), Ukraine, and in Central Germany (*Bitterfeld amber*).

The resin structure contains a polymer matrix and a molecular phase consisting of over 100 organic substances (terpenoids, carboxylic acids, alcohols, and esters) [1]. The polymer matrix is based on polylabdane skeletal, namely co-polymers of communic acid and communol [3]. The resin also contains many fluorescent compounds such as anthracene, 9-methyloanthracene, phenanthrene, retene, acenaphtene, and 2.6 dimethylonaphtalene [4,5]. One of the most characteristic features of Baltic amber is the presence of 3-succinic acid in its structure at a quantity of 3–8% [6]. Most of the succinic acid occurs in Baltic amber in the form of its esters [7,8] or cross-linked with polymers of communic acid and communol [3]. Due to the presence of succinic acid Baltic and Ukrainian ambers belong to class Ia, one of the five major classes [9].

Currently, there are many techniques for studying the properties of amber, such as nuclear magnetic resonance spectroscopy (NMR), pyrolysis gas chromatography with mass spectrometry (Py-GC-MS), microscopic methods, Fourier-transformed infrared spectroscopy (FTIR), Raman spectroscopy, and fluorescence [10,11]. Concerning optical spectroscopy, the most commonly used method is FTIR [1,12,13,14,15,16,17,18,19,20,21,22,23,24]. Recently, the fluorescence method [25,26,27,28,29,30,31,32], which is the most sensitive of all spectroscopic methods, has also been used more and more often. Raman spectroscopy is less frequently used [27,33,34,35,36,37,38,39,40,41]. The spectra in this spectroscopy are complementary to the FTIR technique, but they consist of only a few bands characteristic of C–H and –CH=CH_2_ bonds. In order to improve the attractiveness of Baltic amber, modifications are used, causing a change in color, transparency, and texture. Another procedure is to cover its surface with colored synthetic materials. Therefore, the tests that allow us to distinguish modified materials from natural ones are becoming well-known. This also applies to the identification of samples of scientific value: modern and ancient art, archeological items, or jewelry. The main goal of this work was to examine a series of 25 transmission samples of natural Baltic amber using basic optical spectroscopy methods: absorption in the UV-VIS range, FTIR, scattering Raman, and fluorescence. There are no articles in the literature that analyze materials using all optical spectroscopy methods. In addition, the UV-VIS and fluorescence methods are unique. Many research groups, using, at most, one or two techniques, have been involved in the study of spectroscopic properties of Baltic amber. An important novelty was the introduction of an appropriate method of spectra normalization based not on the comparison of a selected band, but on the spectrum baseline, which allows for reducing the concentration of the tested substance to an identical level and a quantitative comparison of all absorption bands.

Another goal was to compare the spectra of natural and modified amber. This is one of the fundamental problems of amber research and analytical techniques.. Material modification was carried out by heating the material at temperatures of 100–200 °C for 2–8 h. We annealed the samples in a furnace in an air atmosphere at normal pressure. Changes in the FTIR spectra of modified materials were compared within the same samples before modification using the transmission method (inside the sample) and on the surface using the ATR method. The possibilities and usefulness of individual analytical methods for distinguishing aged amber from natural amber were analyzed.

In the literature, the effect of amber modification is usually studied by comparing different materials. This is not appropriate here given the very large dispersion of amber properties throughout individual samples. In addition, measurements are made on finished jewelry products, most often employing the FTIR-ATR method and not using specially prepared plates with an optical, flat surface. The intensity of the reflected radiation depends strongly on the angle of incidence and, in the case of a non-planar surface, the IR spectrum is additionally distorted. Comparative studies of natural amber have shown a large variety of spectra. These differences may not be greater than those resulting from thermal modifications, especially for temperatures below 150 °C. Therefore, optical spectroscopy methods are only useful for identifying large aging changes in amber. Such results are possible when aging studies are conducted on the same samples—before and after modification. This is a different approach than in most of the literature data, where only a few different random samples are analyzed.

## 2. Results

### 2.1. UV-VIS Spectra

**Figure 1:**
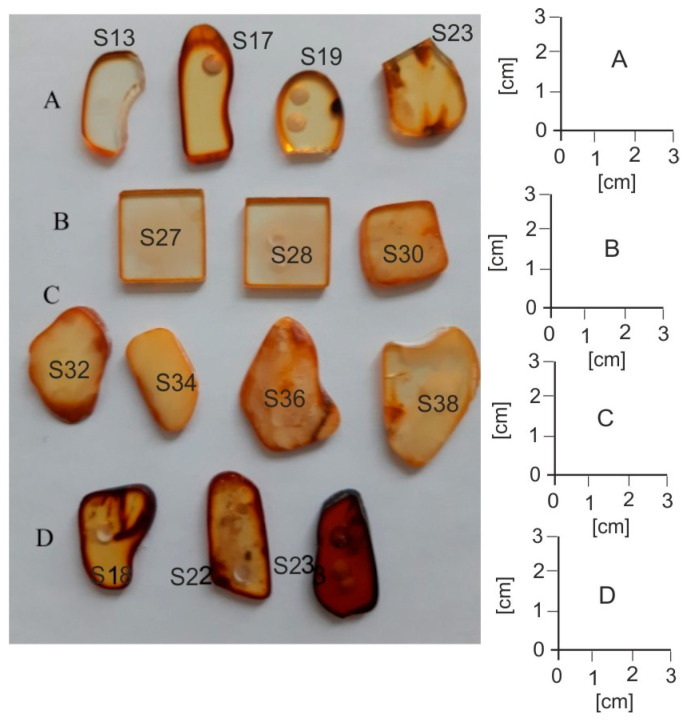
Images of selected amber samples in visible light. (**A**) Transmission samples, (**B**) samples with air bubbles, (**C**) milky, dispersive samples with water and other inclusions, (**D**) transmission samples after heating.

A view of amber plates cut and polished is shown in Figure 1.

Row A shows samples with completely transparent areas and row B samples containing a large number of small air bubbles. Row C contains strongly light-scattering milky samples or those with large inclusions. Row D shows transmissive samples heated at 150 °C (D1), 100 °C (D2), and 200 °C (D3) for 4 h.

**Figure 2:**
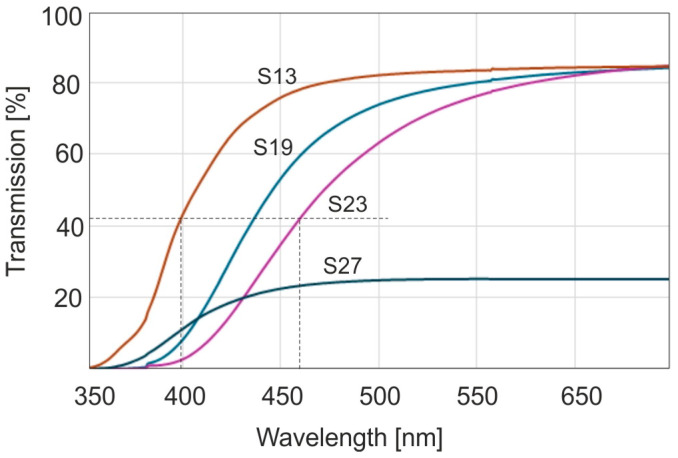
Transmission spectra of selected natural amber samples.

It is clearly visible that the samples are characterized by a large variety of color saturations from very light yellow, described as honey, for naturally occurring samples and brown for thermally aged samples. The color amber is mainly related to the presence of the C=O chromophore. The occurrence of a brown rim about 1–2 mm wide on the edges of each sample is very characteristic. This is the effect of the natural aging of the amber surface in contact with oxygen over millions of years. On some samples there are round marks from sampling the material with a 1 mm drill for FTIR tests. The effect of differences in the color of the samples is best visible in the transmission and absorption spectra in the UV-VIS range (Figure 2).

Sample S13 in Figure 2 represents the amber sample taken from the entire set with the lightest shade of yellow, sample S19 has intermediate characteristics, and sample S23 has the most saturated color. Sample S27 has been taken from row D in Figure 1. These samples are characterized by strong light scattering, hence a significant reduction in transmittance. From the point of view of optics, amber is therefore a high-pass radiation filter. For assessing the position of the transmittance slope, the characteristic value of the radiation wavelength is the value at which the absorbance is equal to half of its maximum value. The dashed lines in the figure indicate full width at half maximum (FWHM). From Figure 2 it follows that these values are located in a range of 400 to 445 nm. This is a very large range of wavelengths responsible for causing so many shades of amber color. The human eye is sensitive to color by about 5 nm with each shift in the spectrum. The range of these differences can be much larger for other samples because there is a huge amount of this resin.

**Figure 3:**
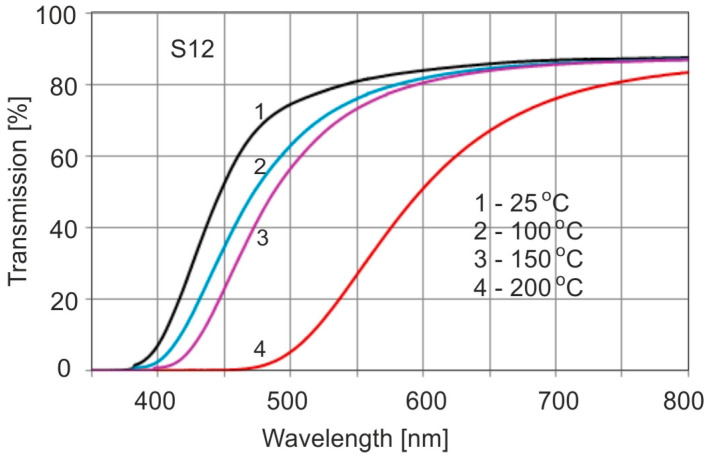
Influence of temperature on the position of the transmission spectrum edges for 4 h heating.

One of the samples was cut into separate parts and each of them was subjected to the study of the effect of temperature on the transmission spectra. Example spectra for different temperatures and heating times for 4 h are shown in Figure 3.

A clear shift in the spectrum towards the long-wavelength side is observed at a temperature of 200 °C. The color of the sample becomes dark brown and resembles the edges of the samples from Figure 1. This shade of color is called cognac. At other temperatures, the color becomes darker than light yellow, but the position of the band is within the limits of many other unaged samples. Hence, it is practically impossible to distinguish thermally aged and unaged samples. For longer heating times, the position of the band edges continues to change. Below a temperature of 100 °C the changes are much slower, and at a temperature of 60 °C they are practically imperceptible.

### 2.2. Amber Fluorescence Studies

**Figure 4:**
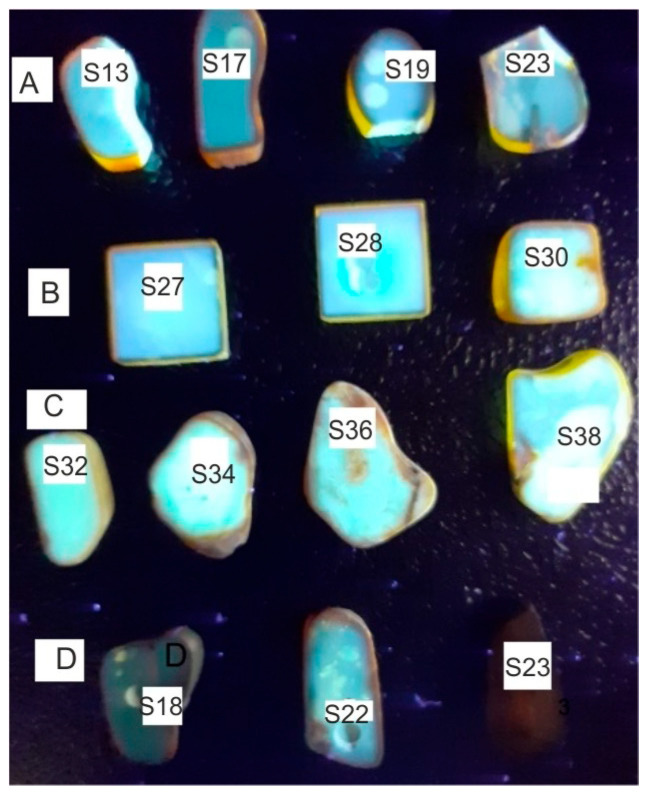
Fluorescence images of Baltic amber samples (samples and scale are the as in Figure 1).

Amber fluorescence is associated with the occurrence of π electronic transitions in double bonds and aromatic compounds. Examples of fluorescence images of the samples from Figure 1 are shown in Figure 4. The samples were excited with a wavelength of 365 nm from Wood’s lamp. It is characteristic that the strongest fluorescence is observed for heavily doped samples. This is the effect of brightening associated with the scattering of both excitation and fluorescence radiation. Light reaches the camera or eyes from many directions. The surface of the frosted sample is therefore brighter compared to the transparent layer. The effect of an apparent increase in the fluorescence level due to scattering is visible, for example, on samples A2 and A3 at the points where the sample was drilled to cut the material for FTIR tests.

Another property of amber is a significant reduction in the fluorescence level of aged samples. At a soak temperature of 200 °C fluorescence disappears completely, after annealing at 150 °C it decreases significantly, and at 100 °C it practically does not change. Brown rims of material on the surfaces of amber do not fluoresce either.

**Figure 5:**
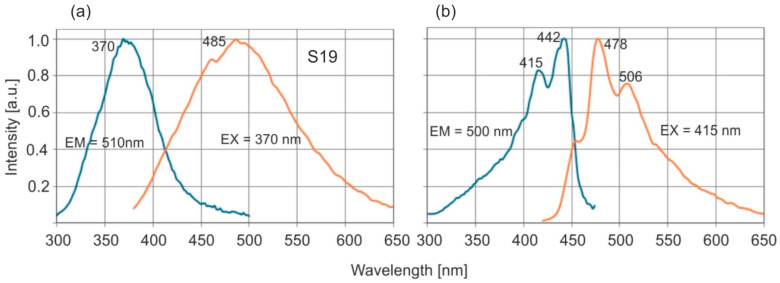
Excitation and emission spectra of (**a**) Baltic amber and (**b**) *Dominican amber*.

Example of excitation and emission spectra of Baltic amber are shown in Figure 5a.

For comparison, the fluorescence spectra of the so-called blue *Dominican amber*, which is a reference point in comparing resins from different regions, are also shown in Figure 5b.

**Figure 6:**
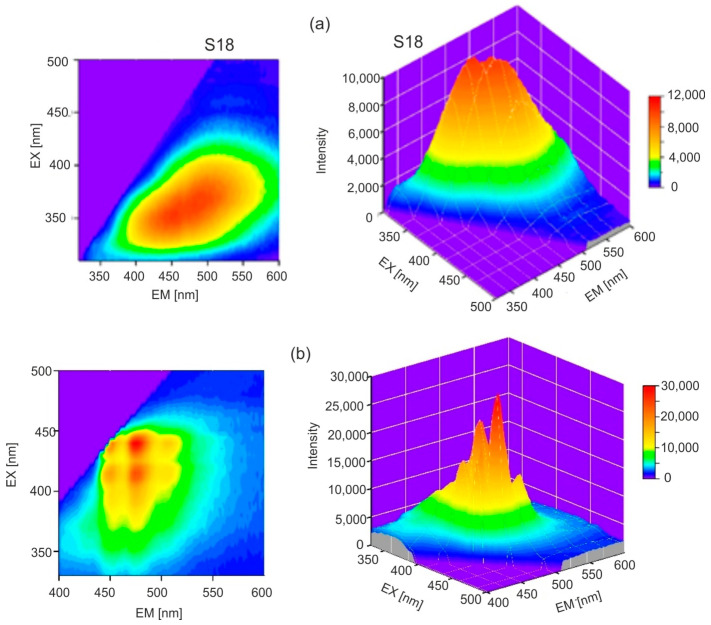
EM–EX maps of (**a**) Baltic amber (S18) and (**b**) *Dominican amber*.

The excitation and emission spectra are symmetrical to each other for both types of amber. In the case of *Dominican amber*, there are distinct, separated absorption bands at 415 and 442 nm and main emission bands at 478 and 506 nm. Pyrene is probably responsible for the occurrence of this fluorescence [31]. The emission bands of Baltic amber are much wider, it lacks a single dominant fluorophore, and a mixture of many compounds is responsible for fluorescence. The full emission characteristics of both ambers are best illustrated by emission–excitation maps (EM–EX), presented in Figure 6.

**Figure 7:**
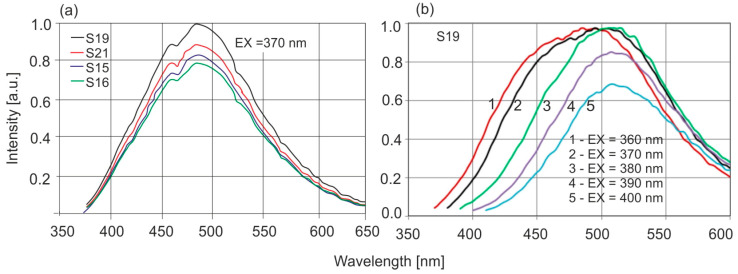
(**a**) Fluorescence of selected Baltic amber samples, (**b**) effect of wavelength on the position of the fluorescence spectrum.

It is clearly visible that, in the case of *Dominican amber*, the excitation and emission bands are much narrower and spectrally clearly separated. Similarly to the position of the absorption band edges, individual Baltic amber samples are characterized by different levels of fluorescence intensity which vary by up to 20% although the position of the wavelength of maximum intensity is constant. The emission characteristics of selected samples at 370 nm excitation are shown in Figure 7a, and Figure 7b shows the effect of the excitation wavelength on the fluorescence spectra.

Maximum fluorescence levels occur for excitation in the range of 360–380 nm. A characteristic shift in the spectrum towards longer wavelengths is observed when the excitation wavelength is increased. For *Dominican amber*, the position of the emission bands during excitation in the entire wavelength range is constant: only the fluorescence intensity changes proportionally to the amount of radiation absorption. This is characteristic of the occurrence of one dominant fluorophore. Clear changes in the position of the fluorescence spectrum of Baltic amber determine such a wide emission range on the EM–EX maps (Figure 6a).

Different levels of fluorescence intensity of individual samples confirm the differences in transmission characteristics (Figure 1). The extent, therefore, to which natural samples can be distinguished from aged samples is an interesting problem since there are significant differences in the emission levels of natural samples.

**Figure 8:**
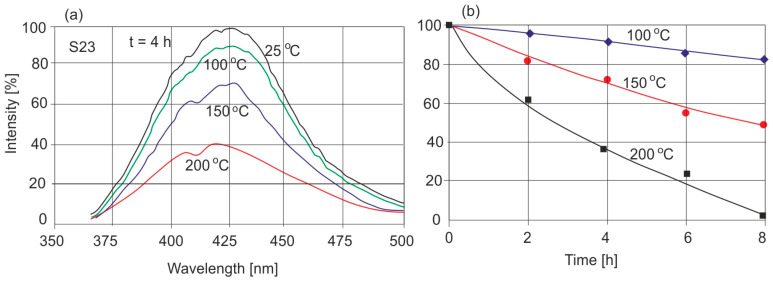
(**a**) Relative change in fluorescence spectrum during 4 h of sample heating and (**b**) kinetics of fluorescence intensity changes over time.

Figure 8a shows an example of the characteristic change in the fluorescence spectrum of the same amber piece (cut into several fragments) when annealed at different temperatures for 4 h, while Figure 8b shows the kinetics of the relative change in the intensity level at different times and temperatures.

**Figure 9:**
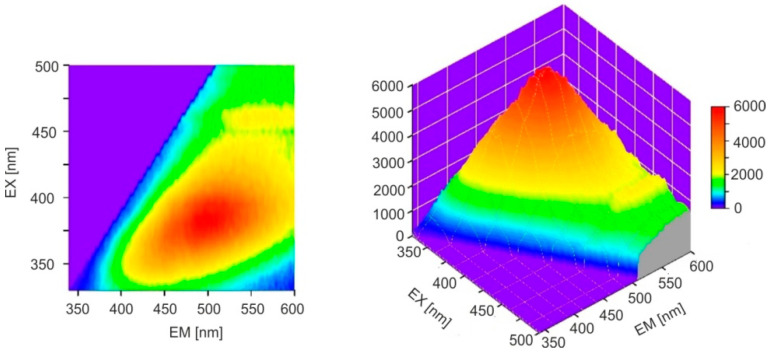
EM–EX map of a sample S23 heated at 150 °C for 4 h.

In a comparison of the fluorescence of different natural samples (Figure 8a) and thermally aged samples, it can be seen that these samples can be distinguished if the heating temperature exceeds 100 °C or when the exposure time is longer (e.g., 4 h for 150 °C). At 200 °C, color changes occur very quickly. These results are in good correlation with transmission measurements (Figure 2). During heating, the EM–EX map is modified, as shown for the sample heated at 150 °C (Figure 9).

A significant difference occurs between Baltic and *Dominican amber*, and there are also visible differences between samples aged at elevated temperatures.

### 2.3. FTIR Studies

**Figure 10:**
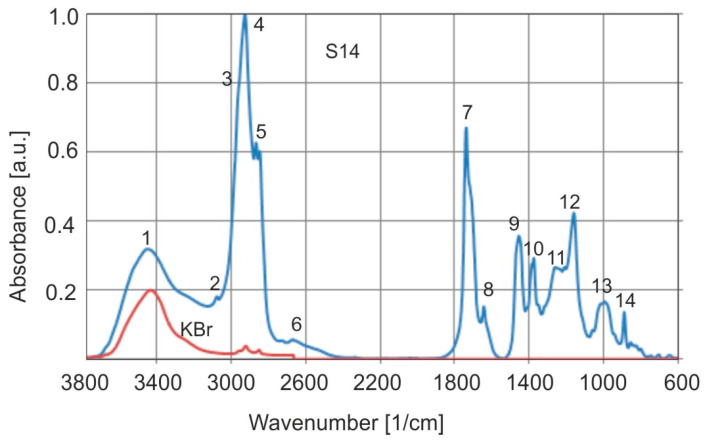
Normalized FTIR absorption spectrum of Baltic amber.

Typical normalized FTIR absorption spectra of Baltic amber are shown in Figure 10, and Table 1 lists the wavenumber values of absorption bands corresponding to oscillatory vibrations of bonds. The KBr spectrum shown in the figure shows that very low absorbance does not have an impact on the height of the amber bands (especially band 4). In that case, there was no need to subtract the absorbance of amber and KBr. In reality, the absorbance of the OH group in amber is low at 0.15–0.2.

**Figure 11:**
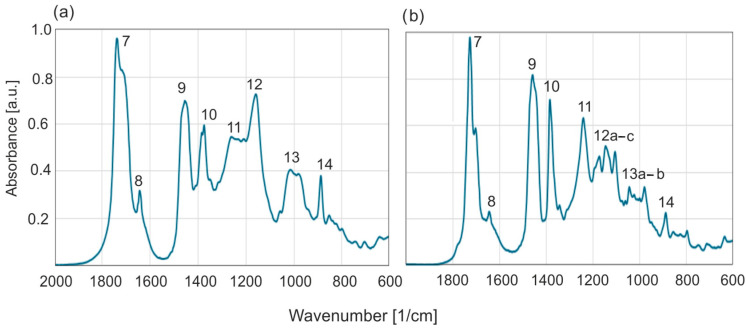
Comparison of FTIR absorption spectra of Baltic (**a**) and Dominican (**b**) amber.

The most characteristic aspect of Baltic amber, regardless of its geographical origin, is the range of wave numbers between 1260 and 1160 cm^−1^, followed by a peak at about 1156 cm^−1^, called the Baltic shoulder [1]. Baltic and *Dominican amber* differ the most within this range (Figure 11).

**Figure 12:**
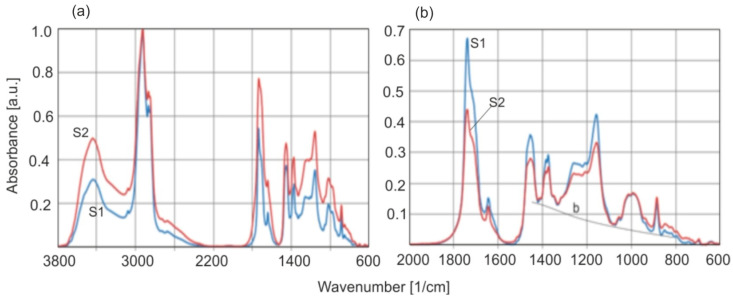
Comparison of absorbance of ambers with the largest difference in the C–H and C=O vibration ratios (**a**) normalization according to the 2869 1/cm band, (**b**) according to the baseline in the range of wavenumbers 1450–600 cm^−1^.

Normalization of transmittance or absorbance spectra is used for qualitative comparison of spectra of different compounds: in this form, they are placed in spectral databases. Absorbance values are normalized relative to their maximum value in the spectrum. The situation becomes more complicated when we want to quantitatively compare the relative absorbance values of individual bands of two different substances. The choice of band is simple when its quantitative share in each spectrum is the same. In publications where the absorbance of ambers is compared, the strongest band around 2925 cm^−1^ associated with asymmetric vibrations ν_as_ (C–H) in (–CH_3_) and ν_as_ (C–H) in (–CH_2_–) were selected for normalization, which is a natural practice. In the studies presented, two methods of normalization of two amber samples, differing in the absorbance ratios of the groups at wave numbers 2925 and 1725 cm^−1^, were compared. Figure 12 shows the absorption characteristics of two amber samples significantly differing in the ratios of the previously mentioned bands (0.78 and 0.58).

Before normalizing the spectra, the absorbance of sample S2 in the C–H band (2869 cm^−1^) was lower compared to sample S1. When normalizing spectra with respect to the C–H vibration, the conclusion is that there are more C=O groups in amber S2 (line red). However, the entire absorption spectrum of S2 in a range of 3800–600 cm^−1^ is artificially raised in this way in relation to the S1 spectrum, including the OH group band (Figure 12a). After normalizing to the baseline (Figure 12b), the absorbance of sample S2 decreased in the entire spectral range in the same ratio, also this applies to the OH band. The absorbances of the samples at 3524 cm^−1^ will be identical and the bands will perfectly overlap if the OH concentration in both samples is identical. The spectrum in Figure 12b is presented only for the range of 2000–600 cm^−1^ to more clearly see the spectral overlap and the course of the baseline.

The assumption of constant absorption in the 2925 cm^−1^ band is obviously incorrect. The only correct conclusive result from such a comparison is the different ratio of the ν_as_ (CH_3_ and CH_2_) bands in relation to ν_as_ (C=O). When we look at the method of normalization in relation to the baseline, the situation is reversed, because the number of groups in amber S2 is clearly smaller in relation to the C=O group in amber S1. So, what is true? The acceptance of variant (b) is supported by the overlap of the baseline in the entire range of 1450–600 cm^−1^, in contrast to variant (a), in which the b1 and b2 baselines diverge. Normalization according to the baseline comes down to the exact equalization of the resin concentrations in both samples. Only in this way can the relative intensities of the remaining bands be compared.

**Figure 13:**
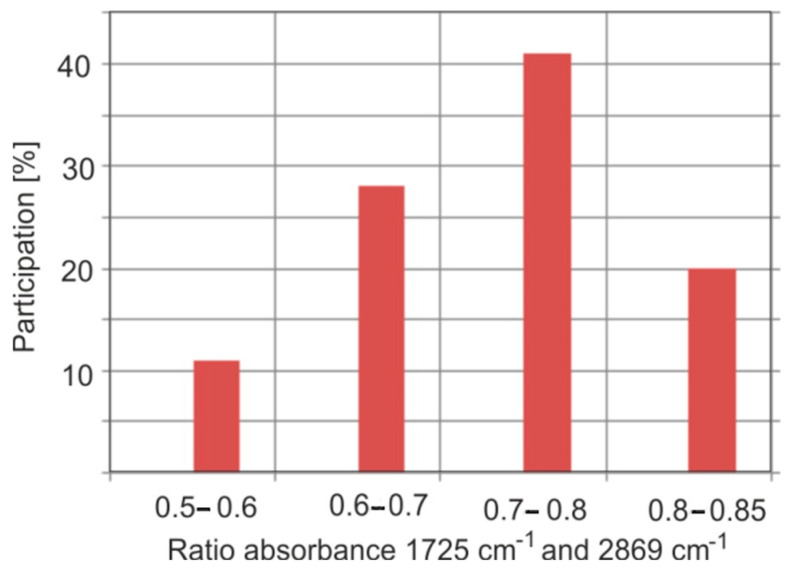
Statistics of absorption band ratios in normalized sample spectra.

The series of samples tested differs greatly in the ratios of the absorption bands of the C–H and C=0 groups. Figure 13 presents the statistics of the ratios of these bands. Only 25 different materials were analyzed: in reality, even greater differences can be expected when the number of samples increases significantly. However, the probability of finding amber with an absorbance ratio of the bands at 1725 and 2869 cm^−1^ in a range of 0.6–0.8 is quite high and is about 70%.

**Figure 14:**
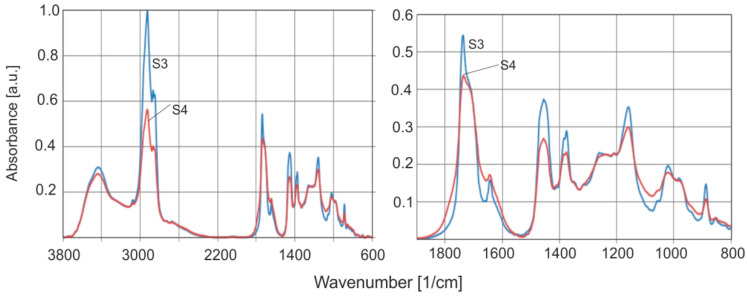
Comparison of absorption spectra of natural amber inside the material (S3) and on the surface (S4).

Figure 14 shows the FTIR spectra of natural amber (S3), and the altered brown rim material (S4) collected from S1. These samples provide an ideal example for investigating the effect of weathering on the absorption spectrum of the material from the beginning of resin formation. The spectra of this altered surface were measured on many samples, and the same result was obtained each time.

The FTIR spectra of both samples show several significant changes. In the case of the surface (S4), a decrease in the intensity of the bands at the C–H and C=O vibrations is observed which can be explained by simple degradation of the material. A clear decrease in the absorbance intensity at C=C bonds (1638 and 886 cm^−1^) is also visible.

Studies on the effect of modified amber on FTIR spectra have been presented in several publications [1,10,14]. The most serious limitation in comparing the absorption properties of natural and aged ambers is the fact that the measurements in the previously cited publications were not made from the same sample before and after aging. As we have shown, the tested series of natural amber samples differ greatly in the ratios of the absorption bands of the C–H and C=O groups (Figure 14).

**Figure 15:**
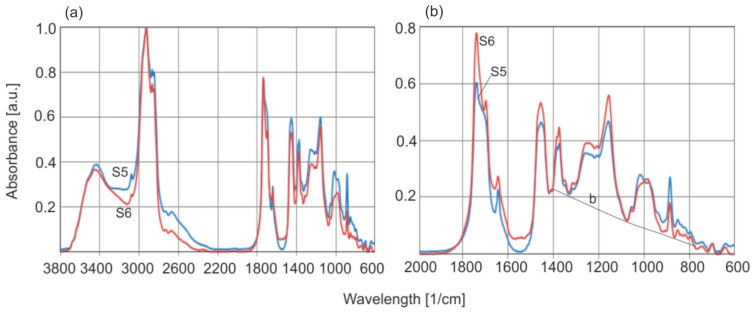
Comparison of absorption spectra of the same sample of natural amber (S5) and thermally aged at 200 °C for 6 h (S6). In Figure 15b the scale has been expanded.

The next step was to conduct studies on the effect of the amber annealing temperature on the FTIR spectrum. Figure 15 shows an example of the change in the spectrum of the sample after heating at 200 °C for 6 h.

**Figure 16:**
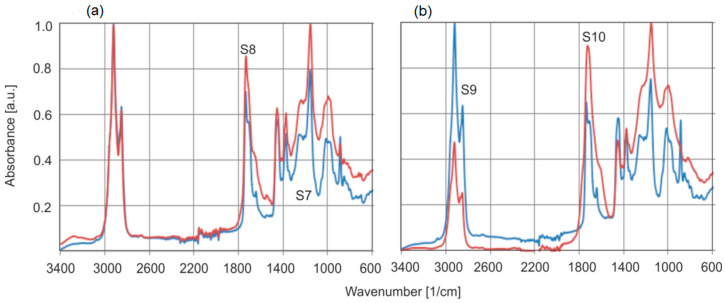
Comparison of FTIR-ATR spectra of natural amber samples (S7, S9) and those modified by temperature: (**a**) T = 150 °C, heating time t = 4 h (S8), (**b**) T = 200 °C, heating time t = 6 h (S10).

The basic change in the spectrum of annealed amber is a decrease in absorbance at the C=C double bond bands (1638 and 886 cm^−1^) and an increase in the C=O carbonyl group band associated with succinic acid esters. However, the changes inside the heated material were minor and smaller than for individual samples from the series of natural amber tested. Greater changes were expected on the surface itself. Figure 16 shows the FTIR-ATR spectra of natural amber samples annealed at temperatures of 150 °C for 2 h and 200 °C for 6 h. Samples S7 and S8 and S9 and S10, respectively, represent the same materials before and after annealing. The spectra were not subjected to the Kramers–Kronig transformation to preserve their original shape.

Heating the samples at moderately high temperatures of 150–200 °C in an air atmosphere leads to drastic changes in the absorbance of individual bands on the sample surfaces. These changes are much greater compared to the changes in the absorbance of double bonds C=C (bands 1638 and 886 cm^−1^) inside the material. The most important change is the increase in the absorbance of the carbonyl group C=O associated with succinic acid esters (1725 cm^−1^).

Generally, spectra collected from the surface are different than those from the bulk.

**Figure 17:**
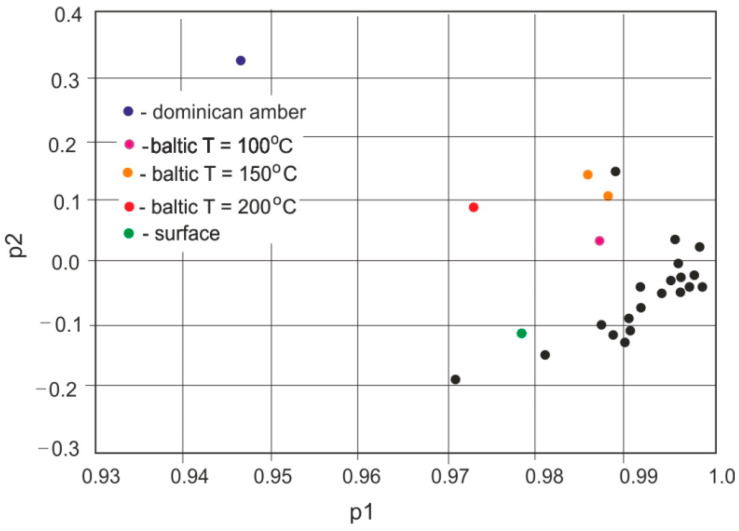
Comparison of absorption properties of ambers using the PCA method.

A comparison of the absorption properties of natural Baltic amber samples, aged Baltic amber, and *Dominican amber* analyzed using the PCA method is presented in Figure 17.

A PCA shows that the spectra of all Baltic amber samples are very different from *Dominican amber*. Furthermore, clear differences can be seen in samples aged thermally on surfaces with smaller changes inside the sample. This is related to the interaction of atmospheric oxygen with the material surface. The position of the PCA map of samples aged at temperatures lower than 150 °C or aged in natural conditions is mixed with the positions of natural samples and it is impossible to determine their modifications on this basis.

For all thermally and naturally aged samples, a clear increase in the absorbance of the C=O group band is observed on the surface, which is consistent with the literature data [1,13]. The chemical interpretation of these changes in the spectra is very difficult. It can be expected that the reduction in the C=C band is related to residual, further polymerization of the material at elevated temperatures. It is difficult, however, to propose any sensible explanation for the increase in the number of C=O bonds. It is possible, for example, that alcohols are oxidized at higher temperatures in the presence of oxygen.

### 2.4. Raman Spectroscopy

**Figure 18:**
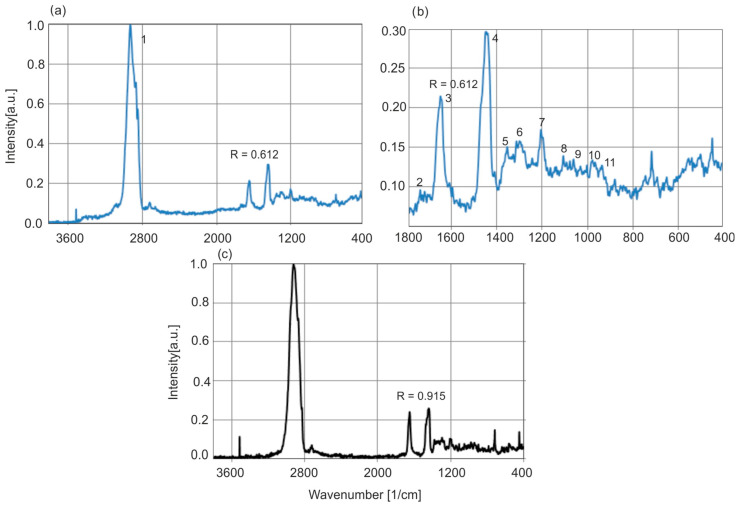
Raman spectrum of Baltic amber (**a**,**b**) and *Dominican amber* (**c**).

Raman scattering spectra of ambers are much poorer in the number of characteristic bands of functional groups of compounds forming the structure of ambers, and hence its usefulness is very limited. This is due to the fact that polar groups (–OH and C=O) have very low intensities in Raman spectroscopy, and only C–H and C=C groups are dominant. Most often, in Raman spectroscopy, the ratio of the intensity of the bands at 1638 and 1455 cm^−1^ associated with C=C and C–H bonds, respectively, is analyzed. But it is valid only in a certain number of cases. On this basis, the degree of polymerization and the related age of the resin can be assessed [34,35,37,39]. It is thought that maturation values below 1 are characteristic of more matured resins, whereas values above are typical for fresh or not so matured resins [34]. Figure 18 shows the Raman spectra of Baltic and *Dominican amber*. Compared to the spectral differences in the IR or fluorescence range, the spectra of both resins differ very little, the only significant difference is the ratio of the C–H and C=C group bands. In the case of the selected samples presented, this ratio for Baltic and *Dominican amber* is 0.625 and 0.915, respectively. On this basis, it could be concluded that Baltic amber is much older than *Dominican amber*. But other authors have questioned the validity of age estimation based solely on band ratios [14]. The older geological age of Baltic amber relative to *Dominican amber* is better supported by stratigraphic and geochronological data from the layers in which they are found.

Table 2 presents the assigned main wavenumber values to individual bonds in the Raman spectra of Baltic amber [34].

**Figure 19:**
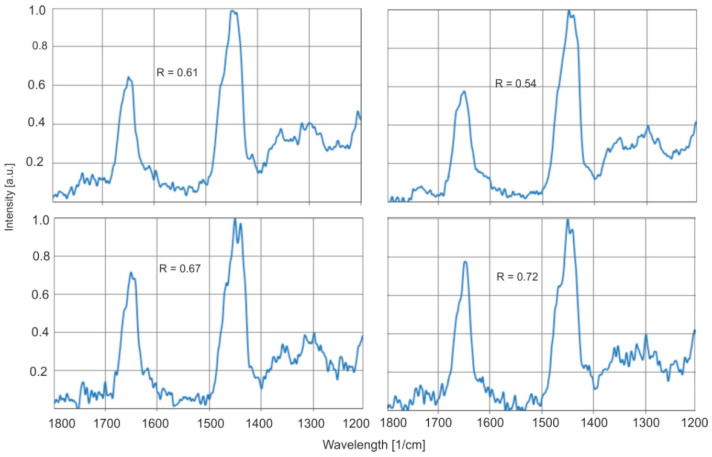
Raman spectra of selected natural amber samples.

Figure 19 shows the Raman spectra of exemplary amber samples.

A series of natural samples differ in the ratio of the C=C and C–H group intensity bands in a range of 0.54–0.80, which confirms the results of previous methods of research on a significant variety of samples.

**Figure 20:**
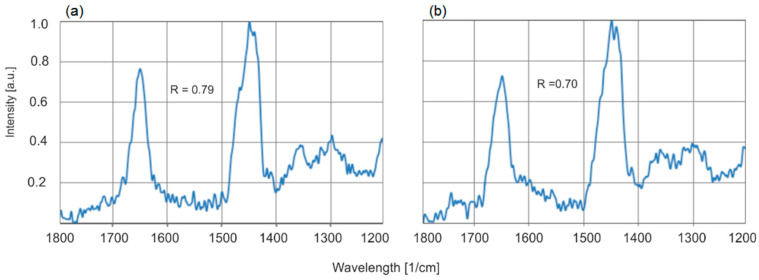
Raman spectra of the same sample of natural amber (**a**) and heated for 6 h at 200 °C (**b**).

For the same samples, a decrease in the number of C=C bonds is observed after annealing which confirms the results obtained by the FTIR method. For amber aged at 150 °C for the same sample, this ratio changes from 0.61 to 0.59, and for 200 °C from 0.79 to 0.70, respectively (Figure 20).

These values are within the range for natural amber, so it is not possible to distinguish modified amber from natural amber based on Raman spectroscopy if we were to compare different samples. The experiment demonstrates only thermal modification. Therefore, it should not be generalized to all types of modifications, such as those involving high pressure or controlled oxidative/inert atmospheres.

At 200 °C, significant changes occur on the amber surface, while the change in the Raman spectrum is small. However, such diagnostics are possible when the characteristics of the surface and inside the sample are compared, because the modification occurs practically at a small depth. It is therefore sufficient to measure the surface spectra, grind the sample surface, polish it, and measure the characteristics under the ground surface again. The method of such an assessment of individual samples based on differences in the Raman spectrum at different depths of the surface was demonstrated in [34]. The authors did not have to grind the surface: they used a confocal microscope equipped with a 785 nm laser for excitation, which enabled measurements at various depths.

## 3. Conclusions

The experiments carried out on 25 different samples of natural Baltic amber show their great diversity. Each type of amber is a mixture of a large number of chemical compounds, very similar or identical, but their proportions in individual samples may be different. The samples were prepared in the form of 2 mm thick flat-parallel plates, which ensure standard and repeatable measurement results. Aging tests were carried out on the same material and spectra were collected before and after aging, which prevents changes related to the diversity of samples.

The position of the transmittance edge of natural amber samples changes in a range of 400–445 nm, which is a significant value and indicates many shades of amber colors. Thermal aging causes a shift in the transmission edge towards longer wavelengths. The effect of a clear shift is visible only from an annealing temperature of 150 °C. For lower temperatures, this shift is within the range of the shift for natural amber. The effect of the edge shift is also visible for lower temperatures, only their much longer heating causes greater changes.

The fluorescence method confirms the diverse properties of natural Baltic amber. Fundamental differences in fluorescence spectra are observed for amber from different geographical locations. Baltic amber contains a mixture of several fluorophores, but there is no dominant one, as in the case of *Dominican amber*, and hence the emission spectrum is broad. The level of fluorescence intensity of individual natural Baltic ambers differs by a maximum of about 20%. With the increase in heating over time, there is a systematic decrease in fluorescence intensity. At a temperature of 200 °C, fluorescence disappears completely after 8 h of heating, which is associated with the decomposition of fluorophores. At moderate temperatures of about 100 °C, the decrease in fluorescence is comparable to the differences in the intensity of natural samples. Changes in fluorescence intensity are accompanied by a decrease in the width of the emission spectrum. The fluorescence method is most useful in assessing the differences between natural and modified amber. Although the fluorescence method seems to be the most promising for distinguishing between natural and modified amber, it should be emphasized that this applies to the ambers we examined that were thermally modified and not modified using other methods.

For these cases of thermal modification to show its effects and the differences, it is enough to have a simple UV radiation source, without the need to measure the spectra, to show changes in the emission intensity on the tested sample and some amber standard. The FTIR method may allow for unambiguous distinction of the geographical origin of natural ambers. However, we did not have samples of amber from other geographical regions, so this conclusion may be too general. We chose *Dominican amber* because the changes in the spectra in relation to Baltic amber are obvious. These changes were related to the differences between natural and thermally aged Baltic amber. A characteristic feature of the FTIR spectra of Baltic amber is the occurrence of a characteristic slope in the range of 1260–1160 cm^−1^. All the Baltic ambers tested have identical absorption bands with changes in the relative intensity of the bands. It has been shown that changes in the absorbance ratio of the bands at 1725 to 2869 cm^−1^ in the normalized spectra lie within a range of 0.5–0.85. Aging tests carried out on the same material (inside the sample) indicate significantly smaller changes in the absorbance values of the samples: only heating them at 200 °C for 8 h causes a significant shift on the PCA map. The FTIR-ATR method indicates that changes on the sample surface caused by interaction with oxygen at elevated temperatures, starting from 150 °C, cause drastic changes in absorption spectra. The absorbance of the C=O group in succinic acid esters increases, which may indicate alcohol oxidation processes. The number of C=C double bonds decreases in aged amber, which indicates the ongoing polymerization process. Raman spectra indicate that Baltic amber samples differ in the absorbance ratio of C–H and unsaturated groups. For thermally aged samples, it was confirmed that the number of C=C bonds decreases, which is consistent with the results obtained using the FTIR method. In general, the usefulness of spectroscopic methods for distinguishing between natural and modified amber at elevated temperatures was confirmed. Significant changes are most visible for temperatures above 150 °C. When examining a sample of amber with small changes based on a single measurement using any spectroscopic method, it is difficult to distinguish whether it is natural or modified. However, such diagnostics are possible when the characteristics of the surface and inside the sample are compared because the modification occurs practically at a small depth. It is therefore sufficient to measure the surface spectra, grind the surface of the sample, polish it, and re-measure the characteristics under the ground surface. Assessment of modification or aging of amber in the case of small changes is possible after comparing the inside of the material and the surface.

## 4. Materials and Methods

The objects of this study were 40 Baltic amber samples collected from the so-called Gdańsk Delta region (the Southern-East part of the Baltic Sea). The world’s largest deposits of Baltic amber are found in this region. The *Dominican amber* came from the La Cumbre region. The samples were collected by geologists.

The resin lumps were cut into 2 mm thick flat-parallel plates and polished. It ensures standard and repeatable measurement results. Aging tests were carried out on the same material and spectra were collected before and after aging, which prevents changes related to the diversity of samples.

The area of the samples ranged from approximately 5 cm^2^ to 8 cm^2^.

Only places with complete transparency and homogeneity were selected for spectral measurements: hence, 15 samples were rejected due to a lack of transparency. On some samples there are round marks from sampling the material with a 1 mm drill for FTIR tests.

The UV-VIS transmission spectra were measured on a Lambda 900 spectrophotometer (Perkin-Elmer, Waltham, MA, USA) with a resolution of 1 nm and averaged over 3 scans.

Fluorescence studies were carried out on an LS 900 spectrometer (Edinburgh Instruments, Livingstone, Scotland). The source of fluorescence excitation was a 450 W xenon lamp. The spectra were recorded at a resolution of 2 nm and the results were averaged over 3 scans. The measurements were taken from polished sample surfaces to avoid the so-called internal filter effect (the effect of absorption of excitation and emitted radiation). Emission–excitation matrices (EM–EX) were measured by changing the excitation wavelength every 5 nm. Three-dimensional and two-dimensional EM–EX plots were prepared using the KyPlot 6.0 program. A Perkin-Elmer GX spectrometer was used for FTIR spectra. FTIR is the most commonly used spectroscopic method in amber studies. Various measurement techniques are used: transmission, reflection (ATR), and scattering. While all of them were available, we chose the classic transmission one. Its basic advantage is that the absorbance value of the measured samples is two orders of magnitude higher than that obtained in ATR measurements, and there is no need to modify the spectra with Kramers–Kronig transforms. In the transmission technique, the spectra measured are very repeatable; the ATR technique is very sensitive to the sample pressure on the diamond window and the shape of the sample surface. The reflection of radiation from the tested surface strongly depends on the angle of incidence of radiation. Hence, samples whose surface is not perfectly flat give additionally distorted spectra. Moreover, in the ATR technique, absorbances are only measured from a depth of about 10–15 μm, and we wanted average measurements from the entire depth of the sample.

The spectra were measured at a resolution of 4 cm^−1^ and averaged over 64 scans. The collected resin samples were powdered, 10 mg of the substance was mixed with 1.0 g of KBr (0.1%), ground in an agate mortar, and 120 mg of the mixture was pressed, obtaining a tablet with a diameter of 7.0 mm and a repeatable thickness of 1.1 mm. Potassium bromide (Sigma-Aldrich, Burlington, Massachusetts, USA) used for disk preparation was of spectral purity. All spectra were baseline-corrected and normalized.

Normalization was not based on comparing the absorbance band with the highest value (e.g., C-H, ν = 2925 cm^−1^), as is typical in the literature [1,14], but consisted in equalizing the concentration of the analyzed samples to the identical concentration by superimposing the spectra so that the baseline in the selected part of the spectrum was the same. Normalization by selected band is convenient for comparing relative band intensities for one spectrum but, in the case of ambers, it cannot be assumed that the concentration of hydrocarbons in each sample is the same. Normalization by baseline allows comparison of C-H band intensities as well.

Principal component analysis (PCA) was performed with the SIMCA-P program from Umetrics (Ulmea, Sweden). PCA is a linear dimensionality reduction technique with applications in exploratory data analysis, visualization, and data preprocessing. The data is linearly transformed into a new coordinate system such that the directions (principal components) capturing the largest variation in the data can be easily identified. The principal components of a collection of points in a real coordinate space are a sequence of p unit vectors, where the i-th vector is the direction of a line that best fits the data while being orthogonal to the first i-1 vectors. A best-fitting line is defined as one that minimizes the average squared perpendicular distance from the points to the line. These directions constitute an orthonormal basis in which different individual dimensions of the data are linearly uncorrelated. When performing PCA, the first principal component of a set of p variables is the derived variable formed as a linear combination of the original variables that explains the most variance. The second principal component explains the most variance in what is left once the effect of the first component is removed, and we may proceed through p iterations until all the variance is explained. In the PCA image, individual points corresponding to the analyzed samples are located close to each other when the spectral properties are similar.

Raman spectra were measured on a Nicolet spectrometer (Nicolet IS50, FTIR, ThermoFisher SCIENTIFIC, Waltham, MA, USA) equipped with an FT-Raman module with an Nd:YAG laser (1064 nm, 0.5 W). The measurement was taken on the polished resin surface using a laser power of 0.25 W and the result was averaged from 200 scans.

ATR-FTIR spectra were measured using a diamond ATR mode in a range of 400–4000 cm^−1^ with a resolution of 4 cm^−1^ and 64 scans, built into the Nicolet instrument.

## Figures and Tables

**Table 1:** Absorption bands and types of oscillations [1].

Band No.	Wave Number	Vibration Type
1	3524	ν(OH)
2	3080	ν(=C-H)
3	2955	ν_as_(C-H) in (-CH_3_)
4	2925	ν_as_(C-H) in (-CH_2_-)
5	2869	ν_s_(C-H) in (-CH_3_) and ν_s_(C-H) in (-CH_2_-)
6	1725	ν(C=O) in esters
7	1700	ν(C=O) in carboxylic acids
8	1642	ν(C=C) in RCH=CH_2_ and RR_1_C=CH_2_
9	1455	δ_as_(C-H) in CH_3_
10	1383	δ_as_(C-H) in CH_3_
11	1239	ν(C-O) in esters, alcohols and carboxylic acids
12	1170	ν(C-O)
13	1044	ν(C-O-H) in alcohols
14	977	γ(C-H) in vinyl (RHC=CH_2_)
15	886	γ(C-H) in viniliden (R_2_C=CH_2_)

**Table 2:** Wavenumbers corresponding to bands in the Raman spectrum [10].

Band No.	Wave Number	Vibration Type
1	2925	ν_as_(–CH_3_) and (–CH_2_)
2	1737	ν(C=O)
3	1645	ν(C=C) non conjugated
4	1450	δ(CH_2_), δ(CH_3_)
5	1442–1304	δ(CH_2_), δ(CH_3_) scissors
6	1304–1298	δ(CH_2_), δ(CH_3_) twisting
7	1205	δ(CCH), δ(C=O)
8	1143	ν(C-C), ν(COH)
9	1003	ν(CC) aromatic
10	976	ρ(CH_2_), ρ(CH_3_)
11	878	ρ (CH_2_)

## Data Availability

Data available after contact with authors.
